# Higher-Order Spreadsheets with Spilled Arrays

**DOI:** 10.1007/978-3-030-44914-8_27

**Published:** 2020-04-18

**Authors:** Jack Williams, Nima Joharizadeh, Andrew D. Gordon, Advait Sarkar

**Affiliations:** ETH Zurich, Zurich, Switzerland; 9 Microsoft Research, Cambridge, UK; 10 University of California, Davis, USA; 11 University of Edinburgh, Edinburgh, UK; 12 University of Cambridge, Cambridge, UK

## Abstract

We develop a theory for two recently-proposed spreadsheet mechanisms: *gridlets* allow for abstraction and reuse in spreadsheets, and build on *spilled arrays*, where an array value spills out of one cell into nearby cells. We present the first formal calculus of spreadsheets with spilled arrays. Since spilled arrays may collide, the semantics of spilling is an iterative process to determine which arrays spill successfully and which do not. Our first theorem is that this process converges deterministically. To model gridlets, we propose the *grid calculus*, a higher-order extension of our calculus of spilled arrays with primitives to treat spreadsheets as values. We define a semantics of gridlets as formulas in the grid calculus. Our second theorem shows the correctness of a remarkably direct encoding of the Abadi and Cardelli object calculus into the grid calculus. This result is the first rigorous analogy between spreadsheets and objects; it substantiates the intuition that gridlets are an object-oriented counterpart to functional programming extensions to spreadsheets, such as sheet-defined functions.

## 1 Introduction

Many spreadsheets contain repeated regions that share the same formatting and formulas, perhaps with minor variations. The typical method for generating each variation is to apply the operations *copy-paste-modify*. That is, the user copies the region they intend to repeat, pastes it into a new location, and makes local modifications to the newly pasted region such as altering data values, formatting, or formulas. A common problem associated with *copy-paste-modify* is that updates to a source region will not propagate to a modified copy. A user must modify each copy manually—a process that is tedious and error-prone.

*Gridlets* [12] are a high-level abstraction for re-use in spreadsheets based on the principle of *live copy-paste-modify*: a pasted region of a spreadsheet can be locally modified without severing the link to the source region. Changes to the source region propagate to the copy.

The *central idea of this paper* is that we can implement gridlets using a formula operator $\mathsf{G}$. If a cell $a$ contains the formula

$$\mathsf{G}(r, a_1, F_1, \ldots, a_n, F_n)$$

then the behaviour is to copy range $r$, modify cells $a_i$ with formulas $F_i$, and paste the computed array in cell $a$ where its elements may be displayed in the cells below and to the right.

Consider the following example:

| | A | B | C |
|---|---|---|---|
| 1 | "*Edge*" | "*Len.*" | |
| 2 | "*a*" | 3 | =B2^2 |
| 3 | "*b*" | 4 | =B3^2 |
| 4 | "*c*" | =SQRT(C4) | =C2 + C3 |

Source sheet

| | A | B | C |
|---|---|---|---|
| 1 | "*Edge*" | "*Len.*" | |
| 2 | "*a*" | 3 | 9 |
| 3 | "*b*" | 4 | 16 |
| 4 | "*c*" | 5 | 25 |

Evaluated sheet

The table computes and displays a Pythagorean triple, with intermediate calculation spread across many cells. To reuse the table a user creates a gridlet by inserting[5] a $\mathsf{G}$ formula in cell A6 as follows.

| | A | B | C |
|---|---|---|---|
| | ⋮ | ⋮ | ⋮ |
| 6 | =G(A1:C4, B2, 7, B3, 24) | | |
| 7 | | | |
| 8 | | | |
| 9 | | | |

Source sheet

| | A | B | C |
|---|---|---|---|
| | ⋮ | ⋮ | ⋮ |
| 6 | "*Edge*" | "*Len.*" | |
| 7 | "*a*" | 7 | 49 |
| 8 | "*b*" | 24 | 576 |
| 9 | "*c*" | 25 | 625 |

Evaluated sheet

The formula in A6 is interpreted as: *compute the source range* A1:C4 *with* B2 *bound to* 7*, and* B3 *bound to* 24*.* The result of the formula is an array corresponding to the computed range which then displays in the grid, emulating a *paste* action. A consequence of this design is that this single formula controls the content of a range of cells, below and to the right; we say that it *spills* into these cells.

Our overall goal is to explain the semantics of the gridlet operator $\mathsf{G}$ using array spilling. Spilling is not new in spreadsheets: both Microsoft Excel and Google Sheets allow a cell to contain a formula that computes an array, and whose computed value then spills into vacant cells below and to the right. While there is a practical precedent for spilling in spreadsheets, there is no corresponding formal precedent from which to derive a semantics for $\mathsf{G}$. This paper therefore proceeds in two parts.

[5] The user may enter this formula either directly, or indirectly via some grid-based interface [12]; details of the user experience are beyond the scope of this paper.

First, we make sense of array spilling and its subtleties. Two formulas spilling into the same cell, or colliding, is one problem. Another problem is a formula spilling into an area on which it depends, triggering a *spill cycle*. Both problems make preserving determinism and acyclicity of spreadsheet evaluation a challenge. We give a semantics of spilling that exploits iteration to determine which arrays spill successfully, and which do not. Our solution ensures that there is at most one array that spills into any address, and that the iteration converges.

Second, we develop three new spreadsheet primitives that implement G when paired with spilled arrays. We present a higher-order spreadsheet calculus, the *grid calculus*, that admits sheets as first-class values and provides operations that manipulate sheet-values. Previous work has drawn connections between spreadsheets and object-oriented programming [5,8,9,15,17], but we give the first direct correspondence by showing that the Abadi and Cardelli object calculus [1] can be embedded in the grid calculus. Our translation constitutes a precise analogy between objects and sheets, and between methods and cells.

In our semantics for gridlets, we make three distinct technical contributions:

- We develop the *spill calculus*, the first formalisation of spilled arrays for spreadsheets. Our first theorem is that the iterative process of spilling we present converges deterministically (Section 4). Our formal analysis of spilled arrays, a feature now available in commercial spreadsheet systems, is a substantial contribution of this work, independent of our gridlet semantics.
- We develop the *grid calculus*, an extension of the spill calculus with three higher-order operators: GRID, VIEW, and UPDATE. These correspond to *copy*, *paste*, and *modify*, and suffice to encode the operator G (Section 5).
- In the course of developing the grid calculus, we realised a close connection between gridlets and object-oriented programming. We make this precise by encoding the Abadi and Cardelli object calculus into the grid calculus. Our second theorem shows the correctness of this encoding (Section 6).

## 2 Challenges of Spilling

In this section we describe the challenges of implementing spilled arrays. We describe core design principles for spreadsheet implementations and then illustrate how spilled arrays challenge these principles.

### 2.1 Design Principles for Spreadsheet Evaluation

Spreadsheet implementations rely on the following two properties to be predictable and efficient.

**Determinism** Evaluation should produce identical output given identical input; this property is exploited for efficient recalculation.

**Acyclicity** Evaluation should not be self-referential. The dependency graph of a spreadsheet should form a directed acyclic graph and no cell should depend on its own value. Creating self-referential formulas cannot be prevented, but violations of acyclicity should be observable and not cause divergence.

Both properties are satisfied by standard spreadsheet implementations, if we exclude a few nondeterministic worksheet functions such as RAND. Throughout this work we consider only deterministic worksheet functions. Given this assumption, spreadsheet formulas constitute a purely functional language, and so evaluation is deterministic. Cell evaluation tracks a *calculating* state for every cell and raises a circularity violation for any cell that depends on its own value.

Spilled arrays pose a challenge for preserving determinism and acyclicity which we illustrate with examples. For the remainder of our technical developments we drop the leading = from formulas. We begin with core terminology.

**Arrays** Spreadsheet arrays are finite two-dimensional matrices that use *one-based* indexing and are non-empty. We denote an $(m, n)$ array literal as

$$\{V_{1,1}, \ldots, V_{1,n}; \ldots; V_{m,1}, \ldots, V_{m,n}\}$$

where (,) delimits the $n$ columns and (;) delimits the $m$ rows. We use $V$ to range over values, which are described in Section 3.

**Spilling** Address $a_r$ $(i, j)$-spills into address $a_t$ iff the value of $a_r$ is an $(m, n)$ array and $a_t$ is $i-1$ rows below and $j-1$ columns right of $a_r$, where $i \in 1..m$ and $j \in 1..n$. In particular, $a_r$ (1,1)-spills into itself.

**Roots, targets, & areas** If $a_r$ $(i, j)$-spills into address $a_t$ we call $a_r$ the *spill root* and $a_t$ a *spill target*. The *spill area* of $a_r$ is the set of its spill targets. The value of $a_t$ is element $(i, j)$ of the array that is the value of $a_r$.

Consider the following example:

| | A | B |
|---|---|---|
| 1 | $\{10, 20\}$ | |
| 2 | | |

Source Sheet

| | A | B |
|---|---|---|
| 1 | 10 | 20 |
| 2 | | |

Evaluated Sheet

Address A1 evaluates to a $(1, 2)$ array and is a spill root with spill area $\{\text{A1}, \text{B1}\}$. Address A1 $(1, 1)$-spills into A1, and $(1, 2)$-spills into B1.

### 2.2 Spill Collisions

Spill collisions can be *static* or *dynamic*, and may interfere with determinism.

*Static Collision* Every cell in a spill area should be *blank* except for the spill root; a blank cell has no formula. A static collision occurs when a spill root spills into another non-blank cell, and we say the non-blank cell is an *obstruction*. The choice to read the value from the obstruction or the spilled value violates determinism. We adopt a simple mechanism used by Excel and Sheets to resolve static spill collisions: the root evaluates to an error value, not an array, and spills nowhere. The ambiguity between reading the obstructing cell's value and the root's spilled value is resolved by preventing the root from spilling—we always read the value from the obstructing cell. Consider the following example:

| | A | B |
|---|---|---|
| 1 | $\{10, 20\}$ | 40 |
| 2 | | $\text{B1} + 2$ |

Source Sheet

| | A | B |
|---|---|---|
| 1 | ERR | 40 |
| 2 | | 42 |

Evaluated Sheet

The address B1 obstructs spill root A1 and consequently address A1 evaluates to an error value, address B1 evaluates to 40, and address B2 evaluates to 42.

*Dynamic Collisions* A dynamic collision occurs when a blank cell is a spill target for two distinct spill roots. Dynamic collisions can be resolved in different ways.

– The conservative approach is to say no colliding spill root spills and each root evaluates to an error.
– The liberal approach is to say that every colliding spill root spills. This approach can be non-deterministic because the spill target obtains its value by choosing one of the multiple colliding spill roots. Google Sheets takes the liberal approach.
– An intermediate approach enforces what we call the *single-spill policy*. One root from the set of colliding roots is permitted to spill and the rest evaluate to an error. This approach can be non-deterministic because there is a choice of which root is permitted to spill. Excel takes the single-spill approach.

Consider the following example that uses the single-spill approach:

| | A | B |
|---|---|---|
| 1 | B2 | $\{3; 4\}$ |
| 2 | $\{1, 2\}$ | |

Source Sheet

| | A | B |
|---|---|---|
| 1 | 2 | ERR |
| 2 | 1 | 2 |

Root A2 wins

| | A | B |
|---|---|---|
| 1 | 4 | 3 |
| 2 | ERR | 4 |

Root B1 wins

Addresses A2 and B1 are spill roots: the former evaluates to an array of size $(1, 2)$ while the latter evaluates to an array of size $(2, 1)$. The value of address A1 depends on which address from the colliding spill roots A2 and B1 are permitted to spill. Arbitrarily selecting which root is permitted to spill violates deterministic evaluation. Sheets and Excel resolve collisions using an ordering that prefers newer formulas. While consecutive evaluations of the same spreadsheet will produce the same result, two syntactically identical spreadsheets constructed in different ways can produce different results. In Section 4 we give a deterministic semantics for spilling that uses a total ordering on addresses to select a single root from a set of colliding roots.

## 2.3 Spill Cycles

A *spill cycle* occurs when the value of a spill root depends on an address in its spill area. Spill cycles violate acyclicity and subtly differ from cell cycles. A cell cycle occurs when the value of a formula in a cell depends on the value of the cell itself. We know that it is never legal for a cell to read its own value and therefore it is possible to eagerly detect cell cycles during evaluation of a cell. In contrast, a spill cycle only occurs if the cell evaluates to an array that is spilled into a range the cell depends on, so it is not possible to detect the cycle until the cell has been evaluated.

We can thus proactively detect cell cycles, but only retroactively detect spill cycles. To see why, let us consider the following example, wherein we assume the definition of a conditional operator $\mathsf{IF}$ that is lazy in the second and third arguments, and the function $\mathsf{INC}$ that maps over an array and increments every number and converts $\epsilon$ to 0, where $\epsilon$ is the value read from a blank cell.

| | A | B |
|---|---|---|
| 1 | 42 | $\mathsf{IF}(\text{A1} = 42, \mathsf{SUM}(\text{B2:B3}), \mathsf{INC}(\text{B2:B3}))$ |
| 2 | | |
| 3 | | |

The evaluation of address B1 returns the sum of the range B2:B3. While the value of B1 depends on the values in the range B2:B3, the sum returns a scalar and therefore no spilling is required.

Consider the case where the value in A1 is changed to 43. The address B1 will evaluate the formula $\mathsf{INC}(\text{B2:B3})$, first by dereferencing the range B2:B3 to yield $\{\epsilon; \epsilon\}$, and then by applying $\mathsf{INC}$ to yield $\{0; 0\}$. The array $\{0; 0\}$ will attempt to spill into the range B1:B2—a range just read from by the formula. The attempt to spill will induce a spill cycle; there is no consistent value that can be assigned to the addresses B1, B2, and B3.

In Section 4 we give a semantics for spilling that uses dynamic dependency tracking to ensure that no spill root depends on its own spill area.

## 3 Core Calculus for Spreadsheets

In this section we present a core calculus for spreadsheets that serves as the foundation of our technical developments.

### 3.1 Syntax

Figure 1 presents the syntax of the core calculus. Let $a$ and $b$ range over A1-style addresses, written $Nm$, composed from a column name $N$ and row index $m$. A column name is a base-26 numeral written using the symbols A..Z. A row index is a decimal numeral written as usual. Let $m$ and $n$ range over positive natural numbers which we typically use to denote row or array indices. We assume a locale in which rows are numbered from top to bottom, and columns from left to right, so that A1 is the top-left cell of the sheet. We use the terms *address* and *cell* interchangeably. Let $r$ range over *ranges* that are pairs of addresses that denote a rectangular region of a grid. Modern spreadsheet systems do not restrict which

| | | | |
|---|---|---|---|
| A1-style column name | $N$ | $::=$ | A \| ... \| Z \| AA \| AB \| ... |
| | $m, n \in$ | | $\mathbb{N}_1$ |
| Address | $a, b$ | $::=$ | $Nm$ |
| Range | $r$ | $::=$ | $a_1{:}a_2$ |
| Value | $V$ | $::=$ | $\epsilon \mid c \mid \mathsf{ERR} \mid \{V_{i,j}{}^{i\in 1..m, j\in 1..n}\}$ |
| Formula | $F$ | $::=$ | $V \mid r \mid f(F_1, \ldots, F_n) \quad (f \text{ function name})$ |
| Sheet | $\mathcal{S}$ | $::=$ | $[a_i \mapsto F_i{}^{i\in 1..n}] \quad (a_i \text{ distinct and no } F_i = \epsilon)$ |
| Grid | $\gamma$ | $::=$ | $[a_i \mapsto V_i{}^{i\in 1..n}] \quad (a_i \text{ distinct})$ |

**Fig. 1.** Syntax for Core Calculus corners of a rectangle are denoted by a range but will automatically normalise the range to represent the top-left and bottom-right corners. We implicitly assume that all ranges are written in the normalised form such that range B1:A2 does not occur; instead, the range is denoted A1:B2.

A value $V$ is either the blank value $\epsilon$, a constant $c$, an error ERR, or a two-dimensional array $\{V_{i,j}{}^{i\in 1..m, j\in 1..n}\}$. We write $\{V_{i,j}{}^{i\in 1..m, j\in 1..n}\}$ as short for array literal $\{V_{1,1}, \ldots, V_{1,n}; \ldots; V_{m,1}, \ldots, V_{m,n}\}$.

Let $F$ range over formulas. A formula is either a value $V$, a range $r$, or a function application $f(F_1, \ldots, F_n)$, where $f$ ranges over names of pre-defined worksheet functions such as SUM or PRODUCT.

Let $\mathcal{S}$ range over sheets, where a sheet is a partial function from addresses to formulas that has finite domain. We write $[]$ to denote the empty map, and we write $\mathcal{S}[a \mapsto F]$ to denote the extension of $\mathcal{S}$ to map address $a$ to formula $F$, potentially shadowing an existing mapping. We do not model the maximum numbers of rows or columns imposed by some implementations. Each finite $\mathcal{S}$ represents an unbounded sheet that is almost everywhere blank: we say a cell $a$ is blank to mean that $a$ is not in the domain of $\mathcal{S}$.

Let $\gamma$ range over grids, where a grid is a partial function from addresses to values that has finite domain. A grid can be viewed as a function that assigns values to addresses, obtained by evaluating a sheet.

### 3.2 Operational Semantics

Figure 2 presents the operational semantics of the core calculus. Auxiliary definitions are present at the top of Figure 2.

*Formula Evaluation* The relation $\mathcal{S} \vdash F \Downarrow V$ means that in sheet $\mathcal{S}$, formula $F$ evaluates to value $V$. A value $V$ evaluates to itself. A function application $f(F_1, \ldots, F_n)$ evaluates to $V$ if the result of applying $[\![f]\!]$ to evaluated arguments is $V$, where $[\![f]\!]$ is the underlying semantics of $f$, a total function on values. A single cell range $a{:}a$ evaluates to $V$ if address $a$ dereferences to $V$. A multiple cell range $a_1{:}a_2$ evaluates to an array of the same dimensions, where each value in the array is obtained by dereferencing the corresponding single cell within the range. We write $\mathsf{size}(a_1{:}a_2)$ to denote the operation that returns the dimensions

$$\begin{aligned}\mathsf{size}(N_1m_1{:}N_2m_2) &= (m_2 - m_1 + 1, N_2 - N_1 + 1)\\ Nm + (i,j) &= (N + j - 1)(m + i - 1)\end{aligned}$$

Formula evaluation: $\mathcal{S} \vdash F \Downarrow V$

$$\frac{}{\mathcal{S} \vdash V \Downarrow V} \qquad \frac{\mathcal{S} \vdash F_i \Downarrow V_i \qquad [\![f]\!](V_1, \dots, V_n) = V}{\mathcal{S} \vdash f(F_1, \dots, F_n) \Downarrow V} \qquad \frac{\mathcal{S} \vdash a\,!\,V}{\mathcal{S} \vdash a{:}a \Downarrow V}$$

$$\frac{a_1 \neq a_2 \qquad \mathsf{size}(a_1{:}a_2) = (m,n) \qquad \forall i \in 1..m, j \in 1..n.\ \mathcal{S} \vdash (a_1 + (i,j))\,!\,V_{i,j}}{\mathcal{S} \vdash a_1{:}a_2 \Downarrow \{V_{i,j}{}^{i\in 1..m, j \in 1..n}\}}$$

Address dereferencing: $\mathcal{S} \vdash a\,!\,V$

$$\frac{\mathcal{S}(a) = F \qquad \mathcal{S} \vdash F \Downarrow V}{\mathcal{S} \vdash a\,!\,V} \qquad \frac{a \notin \mathrm{dom}(\mathcal{S})}{\mathcal{S} \vdash a\,!\,\epsilon}$$

Sheet evaluation: $\mathcal{S} \Downarrow \gamma$

$$\mathcal{S} \Downarrow \gamma \overset{\mathsf{def}}{=} \forall a \in \mathrm{dom}(\mathcal{S}).\ \mathcal{S} \vdash a\,!\,\gamma(a)$$

**Fig. 2.** Operational Semantics for Core Calculus of a range written $(m, n)$, where $m$ is the number of rows, and $n$ is the number of columns. We write $a + (i, j)$ to denote the address offset to the right and below $a$ by $i - 1$ rows and $j - 1$ columns. For example, $a + (1, 1)$ maps to $a$, and $a + (1, 2)$ maps to the address immediately to the right of $a$. Both $\mathsf{size}(a_1{:}a_2)$ and $a + (i, j)$ are defined in Figure 2.

*Address Dereferencing* The relation $\mathcal{S} \vdash a\,!\,V$ means that in sheet $\mathcal{S}$, address $a$ dereferences to $V$. If address $a$ maps to formula $F$ in sheet $\mathcal{S}$, then dereferencing $a$ returns $V$ when $F$ evaluates to $V$. If address $a$ is not in the domain of $\mathcal{S}$ then dereference $a$ returns the blank value $\epsilon$. We make range evaluation and address dereferencing distinct relations to aid our presentation in Section 4.

*Sheet Evaluation* The relation $\mathcal{S} \Downarrow \gamma$ means that sheet $\mathcal{S}$ evaluates to grid $\gamma$ and the relation is defined by point-wise dereferencing of every address in the sheet. Recall the spreadsheet design principles of determinism and acyclicity from Section 2.1. The relations of our semantics are partial functions (as stated in Appendix A of the extended version [21]). As for acyclicity, if there is a cycle where $\mathcal{S}(a) = F$ and evaluation of formula $F$ must dereference cell $a$, then we cannot derive $\mathcal{S} \vdash F \Downarrow V$ for any $V$. Although our calculus could be modified to model a detection mechanism for cell cycles, we omit any such mechanism for the sake of simplicity.

| | | |
|---|---|---|
| Formula | $F ::= \cdots \mid a\#$ | (postfix operator) |
| Dependency set | $\mathcal{D} ::= \{a_1, \ldots, a_n\}$ | |
| Grid | $\gamma ::= [a_i \mapsto (V_i^{\#}, V_i^{!}, \mathcal{D}_i)^{i \in 1..n}]$ | ($a_i$ distinct) |
| Spill permit | $p ::= \checkmark \mid \times$ | |
| Spill oracle | $\omega ::= [a_i \mapsto (m_i, n_i, p_i)^{i \in 1..n}]$ | ($a_i$ distinct) |

**Fig. 3.** Syntax for Spill Calculus (Extends and modifies Figure 1)

## 4 Spill Calculus: Core Calculus with Spilled Arrays

The spill calculus, presented in this section, is the first formalism to explain the semantics of arrays that spill out of cells in spreadsheets. The spill calculus and its convergence, Theorem 1, is our first main technical contribution.

### 4.1 Syntax

Figure 3 presents the extensions and modifications to the syntax of Figure 1; we omit syntax classes that remain unchanged.

Let $F$ range over formulas, extended to include the postfix root operator $a\#$. The root operator $a\#$ evaluates to an array if address $a$ is a *spill root*. Accessing an array via the root operator instead of a fixed-size range is more robust to future edits. For example, consider the sheet $[\mathrm{A1} \mapsto F, \mathrm{B1} \mapsto \mathsf{SUM}(\mathrm{A1}\!:\!\mathrm{A10})]$ where formula $F$ evaluates to a $(10, 1)$ array. If the user modifies $F$ such that the formula evaluates to an array of size $(11, 1)$ then the summation in B1 still applies only to the first ten elements that spill from A1, even if the user intends to sum the whole array. The root operator allows a more robust formulation: $[\mathrm{A1} \mapsto F, \mathrm{B1} \mapsto \mathsf{SUM}(\mathrm{A1}\#)]$. The summation in B1 applies to the entire array that spills from A1, regardless of its size. Section 4.3 shows the full semantics of the root operator.

Let $\mathcal{D}$ range over dependency sets, which denote a set of addresses that a formula bound to an address depends on.

Let $\gamma$ range over grids, which now map addresses to tuples of the form $(V^{\#}, V^{!}, \mathcal{D})$. If $\gamma(a) = (V^{\#}, V^{!}, \mathcal{D})$ then $V^{\#}$ is the pre-spill value obtained by applying the root operator $\#$ to $a$, while $V^{!}$ is the post-spill value obtained by evaluating $a$, and $\mathcal{D}$ is the dependency set required to dereference $a$. Each dereferenced address has both a pre-spill and post-spill value, even if the cell content does not spill. If the pre-spill value is not an array, it cannot spill, and the post-spill value equals the pre-spill value.

Let $p$ range over spill permits, where $\checkmark$ denotes that a root is permitted to spill and $\times$ denotes that it is not.

Let $\omega$ range over spill oracles, which map addresses to tuples of the form $(m, n, p)$. A spill oracle governs how arrays spill in a sheet.

- If $\omega(a) = (m, n, p)$ we expect $a$ to be a spill root for an $(m, n)$ array:
  - If $p = \checkmark$ the contents of $a$ can spill with no obstruction.

$$\text{Let } \mathcal{S} \stackrel{\text{def}}{=} [\text{A1} \mapsto \{7;8\}, \text{B1} \mapsto \mathsf{IF}(\text{A2} = 8, \{9;10\}, 100)]$$

| | A | B |
|---|---|---|
| 1 | {7; 8} | 100 |
| 2 | | |

Round 1: $\omega_1 = []$

| | A | B |
|---|---|---|
| 1 | 7 | {9; 10} |
| 2 | 8 | |

Round 2: $\omega_2 = [\text{A1} \mapsto (2, 1, \checkmark)]$

| | A | B |
|---|---|---|
| 1 | 7 | 9 |
| 2 | 8 | 10 |

Round 3: $\omega_3 = [\text{A1} \mapsto (2, 1, \checkmark), \text{B1} \mapsto (2, 1, \checkmark)]$

**Fig. 4.** Example Spill Iteration

- If $p = \times$ then $a$ cannot spill because either a formula obstructs the spill area, or another spill root will spill into the area.

Oracles track the size of each spilled array so we can find the spill root $a$ of any spill target, and hence obtain the value for a spill target by dereferencing $a$.

### 4.2 Spill Oracles and Iteration

As discussed in Section 2.2, spill collisions have the potential to introduce non-determinism if not handled appropriately. Our solution is to evaluate a sheet in a series of rounds, each determined by a *spill oracle*. Given a sheet, a grid is induced by evaluating the sheet and using the oracle to deterministically predict how each root spills. A discrepancy could be a new spill root the oracle missed, or an existing spill root with dimensions differing from the oracle. If any discrepancies are found we compute a new oracle, and start a new round. Iteration halts when the oracle is *consistent* with the induced grid. The notion of a consistent oracle is defined in Section 4.4. We can view the iteration as a sequence of $n$ oracles where only the final oracle is consistent:

$$[] = \omega_1 \longrightarrow \omega_2 \longrightarrow \cdots \longrightarrow \omega_n \text{ and } \omega_n \text{ is consistent}$$

Consider the example in Figure 4. At the top we show the bindings of the sheet; at the bottom we show the oracle and induced grid for each round of spilling.

We define the initial spill oracle as $\omega_1 = []$ and in the first round the oracle is empty. An empty oracle anticipates no spill roots and therefore no roots are permitted to spill. The array in A1 remains collapsed and B1 evaluates using the false branch. Once the sheet has been fully evaluated we determine that $\omega_1$ was not a consistent prediction because there is an array in A1 with no corresponding entry in $\omega_1$. We compute a new oracle that determines that A1 is allowed to spill because the area is blank. We define the new oracle as $\omega_2 = [\text{A1} \mapsto (2, 1, \checkmark)]$.

In the second round the root A1 is permitted to spill by the oracle and as a consequence B1 now evaluates to the array {9; 10}—this array is not anticipated by the oracle and remains collapsed. Once the sheet has been fully evaluated we determine that $\omega_2$ was not a consistent prediction because there is an array in

B1 with no corresponding entry in $\omega_2$. We compute a new oracle that determines that B1 is allowed to spill because the area is blank in the grid induced by $\omega_2$. We define the third oracle as $\omega_3 = [\mathrm{A1} \mapsto (2,1,\checkmark), \mathrm{B1} \mapsto (2,1,\checkmark)]$.

In the third and final round the root A1 is permitted to spill by the oracle and B1 evaluates to the array $\{9;10\}$. This time the oracle anticipates the root in B1 and permits the array to spill. Once the sheet has been fully evaluated we determine that $\omega_3$ is a consistent prediction because the spill roots A1 and B1 are contained in the oracle. The iteration is the sequence of three oracles:

$$[] \longrightarrow [\mathrm{A1} \mapsto (2,1,\checkmark)] \longrightarrow [\mathrm{A1} \mapsto (2,1,\checkmark), \mathrm{B1} \mapsto (2,1,\checkmark)]$$

*Spill Rejection* Spill oracles explicitly track the anticipated size of the array to ensure that spill rejections based on incorrect dimensions can be corrected. Consider the following example:

| | A | B | C |
|---|---|---|---|
| 1 | | $\mathsf{IF}(\mathrm{C2} = 2, \{10;20\}, \{10;20;30\})$ | $\{1;2\}$ |
| 2 | | | |
| 3 | $\{1,2,3\}$ | | |

After the first round using an empty spill oracle there are three spill roots: $\mathrm{A3} = \{1,2,3\}$, $\mathrm{B1} = \{10;20;30\}$, and $\mathrm{C1} = \{1;2\}$. There is sufficient space to spill C1 but only space to spill one of A3 and B1; the decision is resolved using the total ordering on addresses. Suppose that we allow A3 to spill such that the new oracle is: $[\mathrm{A3} \mapsto (1,3,\checkmark), \mathrm{B1} \mapsto (3,1,\times), \mathrm{C1} \mapsto (2,1,\checkmark)]$.

After the second round we find that address B1 returns an array of a smaller size because the root C1 spills into C2. Previously we thought B1 was too big to spill but with the new oracle we find there is now sufficient room; by explicitly recording the anticipated size it is possible to identify cases that require further refinement. We compute the new oracle $[\mathrm{A3} \mapsto (1,3,\checkmark), \mathrm{B1} \mapsto (2,1,\checkmark), \mathrm{C1} \mapsto (2,1,\checkmark)]$ that is consistent.

An interesting limitation arises if the total ordering places B1 before A3, which we discuss in Section 4.6.

## 4.3 Operational Semantics

Figure 5 presents the operational semantics for the spill calculus. The key additions to the relations for formula evaluation and address dereferencing are an oracle $\omega$ that is part of the context, and a dependency set $\mathcal{D}$ that is part of the output. We discuss each relation in turn and focus on the extensions and modifications from Figure 2. Auxiliary definitions are present at the top of Figure 5.

*Formula Evaluation:* $\mathcal{S}, \omega \vdash F \Downarrow V, \mathcal{D}$ The spill oracle $\omega$ is not inspected by the relation but is threaded through the definition. Dependency set $\mathcal{D}$ denotes the transitive dependencies required to evaluate $F$. Evaluating a value or function application is as before, except we additionally compute the dependencies of the

$$\begin{aligned}
\mathsf{owners}(\omega, a) &= \{(a_r, i, j) \mid \omega(a_r) = (m, n, \checkmark) \text{ and } a_r + (i, j) = a \text{ and } (i, j) \leq (m, n)\} \\
\mathsf{area}(a, m, n) &= \{\, a + (i, j) \mid \forall i \in 1..m, \forall j \in 1..n \,\} \\
\mathsf{size}(V) &= \begin{cases} (m, n) & \text{if } V = \{V_{i,j}{}^{i \in 1..m, j \in 1..n}\} \\ \bot & \text{otherwise} \end{cases}
\end{aligned}$$

Formula evaluation: $\mathcal{S}, \omega \vdash F \Downarrow V, \mathcal{D}$

$$\frac{}{\mathcal{S}, \omega \vdash V \Downarrow V, \varnothing} \qquad \frac{\mathcal{S}, \omega \vdash F_i \Downarrow V_i, \mathcal{D}_i \qquad [\![f]\!](V_1, \ldots, V_n) = V}{\mathcal{S}, \omega \vdash f(F_1, \ldots, F_n) \Downarrow V, \bigcup_{i=1}^{n} \mathcal{D}_i}$$

$$\frac{\mathcal{S}, \omega \vdash a \,!\, V^{\#}, V^{!}, \mathcal{D}}{\mathcal{S}, \omega \vdash a\# \Downarrow V^{\#}, \mathcal{D} \cup \{a\}} \qquad \frac{\mathcal{S}, \omega \vdash a \,!\, V^{\#}, V^{!}, \mathcal{D}}{\mathcal{S}, \omega \vdash a{:}a \Downarrow V^{!}, \mathcal{D} \cup \{a\}}$$

$$\frac{\begin{array}{c} a_1 \neq a_2 \\ \mathsf{size}(a_1{:}a_2) = (m, n) \qquad \forall i \in 1..m, j \in 1..n.\ \ \mathcal{S}, \omega \vdash a_1 + (i, j) \,!\, V^{\#}_{i,j}, V^{!}_{i,j}, \mathcal{D}_{i,j} \end{array}}{\mathcal{S}, \omega \vdash a_1{:}a_2 \Downarrow \{V^{!}_{i,j}{}^{i \in 1..m, j \in 1..n}\}, \bigcup_{i,j=1,1}^{m,n} \mathcal{D}_{i,j} \cup \{a_1 + (i, j)\}}$$

Address dereferencing: $\mathcal{S}, \omega \vdash a \,!\, V^{\#}, V^{!}, \mathcal{D}$

$$\frac{\mathsf{owners}(\omega, a) = \varnothing \qquad a \notin \mathrm{dom}(\omega) \qquad \mathcal{S}(a) = F \qquad \mathcal{S}, \omega \vdash F \Downarrow V, \mathcal{D}}{\mathcal{S}, \omega \vdash a \,!\, V, V, \mathcal{D}} \quad (1)$$

$$\frac{\mathsf{owners}(\omega, a) = \varnothing \qquad a \notin \mathrm{dom}(\omega) \qquad a \notin \mathrm{dom}(\mathcal{S})}{\mathcal{S}, \omega \vdash a \,!\, \epsilon, \epsilon, \varnothing} \quad (2)$$

$$\frac{\mathsf{owners}(\omega, a) = \varnothing \qquad \omega(a) = (m, n, \times) \qquad \mathcal{S}(a) = F \qquad \mathcal{S}, \omega \vdash F \Downarrow V, \mathcal{D}}{\mathcal{S}, \omega \vdash a \,!\, V, \mathsf{ERR}, \mathcal{D}} \quad (3)$$

$$\frac{\begin{array}{c} (a_r, i, j) \in \mathsf{owners}(\omega, a) \qquad \omega(a_r) = (m, n, \checkmark) \qquad \mathcal{S}(a_r) = F \\ \mathcal{S}, \omega \backslash a_r \vdash F \Downarrow V, \mathcal{D} \qquad \mathsf{size}(V) = (m, n) \qquad \mathsf{area}(a_r, m, n) \cap \mathcal{D} = \varnothing \end{array}}{\mathcal{S}, \omega \vdash a \,!\, (a = a_r \,?\, V : \epsilon), V_{i,j}, \mathcal{D}} \quad (4)$$

$$\frac{\begin{array}{c} (a_r, i, j) \in \mathsf{owners}(\omega, a) \\ \omega(a_r) = (m, n, \checkmark) \qquad \mathcal{S}(a_r) = F \qquad \mathcal{S}, \omega \backslash a_r \vdash F \Downarrow V, \mathcal{D} \qquad \mathsf{size}(V) \neq (m, n) \end{array}}{\mathcal{S}, \omega \vdash a \,!\, (a = a_r \,?\, V : \epsilon), (a = a_r \,?\, V : \epsilon), (a = a_r \,?\, \mathcal{D} : \varnothing)} \quad (5)$$

Sheet evaluation: $\mathcal{S}, \omega \Downarrow \gamma$

$$\mathcal{S}, \omega \Downarrow \gamma \stackrel{\mathsf{def}}{=} \forall a \in \mathrm{dom}(\mathcal{S}).\ \mathcal{S}, \omega \vdash a \,!\, \gamma(a)$$

**Fig. 5.** Operational Semantics for Spill Calculus formula. The dependency set required to evaluate a value is $\varnothing$. The dependency set required to evaluate a function application is the union of the dependencies of the arguments. Evaluating a root operation $a\#$ dereferences $a$ and returns the pre-spill value $V^{\#}$. The dependency set required to evaluate a root operation $a\#$ is the dependency set required to dereference $a$ and the address $a$ itself. Evaluating a single cell range $a{:}a$ dereferences $a$ and returns the post-spill value $V^{!}$. The dependency set required to evaluate a single cell range $a:a$ is the dependency set required to dereference $a$ and the address $a$ itself. Evaluating a multiple cell range $a_1{:}a_2$ returns an array of the same dimensions, where each value in the array is obtained by dereferencing the corresponding single cell and extracting the post-spill value. The dependency set required to evaluate a multiple cell range is the dependency set required to dereference every address in the range, and the range itself.

*Address dereferencing* The relation $\mathcal{S}, \omega \vdash a \,!\, V^{\#}, V^{!}, \mathcal{D}$ means that in sheet $\mathcal{S}$ with oracle $\omega$, address $a$ dereferences to pre-spill value $V^{\#}$ and post-spill value $V^{!}$, and depends upon the addresses in $\mathcal{D}$. Five rules govern address dereferencing, based on spill oracle $\omega$ and *owners* set $\mathsf{owners}(\omega, a)$.

The set $\mathsf{owners}(\omega, a)$ is key to the operational semantics and denotes the set of owners for address $a$. If a tuple $(a_r, i, j)$ is in the set $\mathsf{owners}(\omega, a)$, we say $a_r$ *owns* $a$, meaning that $a_r$ is a spill root that we expect to spill into address $a$, and that $a$ is offset from $a_r$ by $i-1$ rows and $j-1$ columns. Hence, to dereference $a$ we must first compute the root $a_r$ and extract the $(i,j)^{th}$ spilled value from the root array. Our definition allows an address to own itself, denoted $(a, 1, 1) \in \mathsf{owners}(\omega, a)$, and does not preclude an address having multiple owners, violating the *single-spill policy*. We enforce the single-spill policy in our technical results using an additional well-formedness condition on oracles, defined in Section 4.5.

Rule (1) applies when the address has no owner, the address is not a spill root, and the address has a formula binding in $\mathcal{S}$. The pre-spill and post-spill values are the value obtained by evaluating the bound formula.

Rule (2) applies when the address has no owner, the address is not a spill root, and the address has no formula binding in $\mathcal{S}$. The pre-spill and post-spill values are the blank value $\epsilon$ and the dependency set is empty. Rules (1) and (2) correspond to the address dereferencing behaviour described in the core calculus (Section 3) which is lifted to the new relation.

Rule (3) rule applies when the address is a spill root and the root is not permitted to spill. The pre-spill value is the value obtained by evaluating the bound formula; the post-spill value is an error value. If the address has no bound formula then the relation is undefined.

Rules (4) and (5) apply when an address with an *owner* is dereferenced. The owner $a_r$ is omitted from the spill oracle before evaluating the associated formula, denoted by $\mathcal{S}, \omega \backslash a_r \vdash F \Downarrow V, \mathcal{D}$. This prevents cycles when the oracle incorrectly expects the root to spill, but the root does not, and instead depends on the expected spill area. For example, $\mathrm{B1} = \mathsf{SUM}(\mathrm{B2{:}B3})$ and $\omega = [\mathrm{B1} \mapsto (3, 1, \checkmark)]$. The address B1 owns B2 according to $\omega$, therefore dereferencing address B2 requires dereferencing B1, which in-turn depends on B2. If we did not remove

B1 from $\omega$ when evaluating the formula bound to B1 we would create a cycle. We remove B1 from $\omega$ so that when formula SUM(B2:B3) dereferences B2 a blank value is returned. Genuine spill cycles are detected post-dereferencing using the dependency set.

Rule (4) applies when the address has an owner and the formula bound to the owner evaluates to an array of the expected size according to $\omega$. This rule is only defined when the intersection of the spill root's dependencies and its spill area is empty, preventing spill cycles. The pre-spill value is obtained using the conditional operator $a = a_r \,?\, V : \epsilon$. When the dereferenced cell is the root then the value is the root array, otherwise the value is blank. The post-spill value is obtained by indexing into the root array at the $(i, j)^{th}$ position.

Rule (5) applies when the address has an owner and the formula bound to the owner *does not* evaluate to an array of the expected size according to $\omega$. In this case there is no attempt to spill as the oracle is incorrect. When the dereferenced address is the root then the pre-spill and post-spill values are obtained from the formula, otherwise the pre-spill and post-spill values are blank.

*Sheet evaluation:* $\mathcal{S}, \omega \Downarrow \gamma$ Sheet evaluation in the spill calculus accepts a spill oracle, but is otherwise unchanged from sheet evaluation in the core calculus. The computed grid only contains the value of addresses with a bound formula, and does not include the value of any blank cells that are in a spill area. In contrast, a spreadsheet application would display the value for all addresses, including those within a spill area. Obtaining this view can be done by dereferencing every address in the viewport using the sheet and oracle.

### 4.4 Oracle Refinement

We have shown how to compute a grid given a sheet and oracle, but we have not considered the accuracy of the predictions provided by the oracle. In Section 4.2 we informally describe an iterative process to refine an oracle from a computed grid; in this section we give the precise semantics of oracle refinement. Figure 6 presents the full definition of oracle refinement.

*Consistency* The relation $\gamma \models \omega$ states that grid $\gamma$ is consistent with oracle $\omega$. A grid is consistent if every address is consistent, written $\gamma \models_a \omega$. An address $a$ is consistent in $\gamma$ and $\omega$ if, and only if, the grid and oracle agree on the size of the value at address $a$. Consistency tells us that the oracle has correctly predicted the location and size of every spill root in the grid, and has not predicted any spurious roots.

*Refinement* The function $\mathsf{refine}(\mathcal{S}, \omega, \gamma)$ takes an inconsistent oracle and returns a new oracle that is refined using the computed grid. The function is defined as follows. First, start with subset $\omega_{\text{ok}}$ of $\omega$ that is consistent with $\gamma$. Second, collect the remaining unresolved spill roots in $\gamma$, denoted $\gamma_r$. Finally, recursively select the smallest address in $\gamma_r$ according to a total order on addresses, determining whether the root is permitted to spill and adding the permit to the accumulating

$$\gamma \models_a \omega \stackrel{\mathsf{def}}{=} \forall m,n,p.\ (\omega(a) = (m,n,p)) \Leftrightarrow \exists V^{\#}, V^{!}, \mathcal{D}.\ (\gamma(a) = (V^{\#}, V^{!}, \mathcal{D}) \wedge \mathsf{size}(V^{\#}) = (m,n))$$
$$\gamma \models \omega \stackrel{\mathsf{def}}{=} \forall a.\ \gamma \models_a \omega$$

$$\mathsf{refine}(\mathcal{S}, \omega, \gamma) = \mathsf{decide}(\mathcal{S}, \omega_{\text{ok}}, \gamma_r) \text{ where}$$

$$\omega_{\text{ok}} = \{a \mapsto (m,n,p) \in \omega \mid \gamma \models_a \omega\}$$
$$\gamma_r = \{a \mapsto (V^{\#}, V^{!}, \mathcal{D}) \in \gamma \mid \exists m,n.\ \mathsf{size}(V^{\#}) = (m,n) \text{ and } a \notin \text{dom}(\omega_{\text{ok}})\}$$

$$\mathsf{decide}(\mathcal{S}, \omega, []) = \omega$$
$$\mathsf{decide}(\mathcal{S}, \omega, \gamma[a \mapsto (V^{\#}, V^{!}, \mathcal{D})]) = \mathsf{decide}(\mathcal{S}, \omega[a \mapsto (m,n,p)], \gamma)$$
where $a$ is the least element in $\text{dom}(\gamma)$ and $\mathsf{size}(V^{\#}) = (m,n)$
$$p = \begin{cases} \checkmark & \text{if } \forall a_t \in \mathsf{area}(a,m,n).\ a \neq a_t \Rightarrow a_t \notin \text{dom}(\mathcal{S}) \text{ and } \mathsf{owners}(\omega, a_t) = \varnothing \\ \times & \text{otherwise} \end{cases}$$

Spill iteration: $\omega \longrightarrow_{\mathcal{S}} \omega'$

Final oracle: $\mathcal{S} \vdash \omega\ \mathsf{final}$

$$\frac{\mathcal{S}, \omega \Downarrow \gamma \qquad \gamma \not\models \omega \qquad \mathsf{refine}(\mathcal{S}, \omega, \gamma) = \omega'}{\omega \longrightarrow_{\mathcal{S}} \omega'} \qquad \frac{\mathcal{S}, \omega \Downarrow \gamma \qquad \gamma \models \omega}{\mathcal{S} \vdash \omega\ \mathsf{final}}$$

Final sheet evaluation: $\mathcal{S} \Downarrow \gamma$

$$\mathcal{S} \Downarrow \gamma \stackrel{\mathsf{def}}{=} [] \longrightarrow_{\mathcal{S}}^{*} \omega \text{ and } \mathcal{S} \vdash \omega\ \mathsf{final} \text{ and } \mathcal{S}, \omega \Downarrow \gamma$$

**Fig. 6.** Oracle Refinement oracle. A root is permitted to spill if the potential spill area is blank (excluding the root itself) and each address in the spill area has no owner, thereby preserving the single-spill policy.

*Spill iteration* The relation $\omega \longrightarrow_{\mathcal{S}} \omega'$ denotes a single iteration of oracle refinement. When a computed grid is not consistent with the spill oracle that induced it, written $\gamma \not\models \omega$, a new oracle is produced using function $\mathsf{refine}(\mathcal{S}, \omega, \gamma)$. We write $\longrightarrow_{\mathcal{S}}^{*}$ for the reflexive and transitive closure of $\longrightarrow_{\mathcal{S}}$.

*Final oracle* The relation $\mathcal{S} \vdash \omega\ \mathsf{final}$ states that oracle $\omega$ is final for sheet $\mathcal{S}$, and is valid when the grid induced by $\omega$ is consistent with $\omega$.

*Final sheet evaluation* The relation $\mathcal{S} \Downarrow \gamma$ denotes the evaluation of sheet $\mathcal{S}$ to grid $\gamma$ which implicitly refines an oracle to a final state. The process starts with an empty oracle [] and iterates until a final oracle is found.

### 4.5 Technical Results

This section presents the main technical result of the spill calculus: that iteration of oracle refinement converges for well-behaved sheets. We begin with preliminary definitions and results.

To avoid ambiguous evaluation every spill area must be disjoint and unobstructed; an oracle is *well-formed* if it predicts non-blank spill roots, and predicts disjoint and unobstructed spill areas, defined below:

**Definition 1 (Well-formed oracle).** *We write $\mathcal{S} \vdash \omega$ $\mathsf{wf}$ if oracle $\omega$ is well-formed for sheet $\mathcal{S}$. An oracle $\omega$ is well-formed if for all addresses $a$ the following conditions are satisfied:*

1. *If $a \notin \mathrm{dom}(\mathcal{S})$ then $a \notin \mathrm{dom}(\omega)$.*
2. $|\mathsf{owners}(\omega, a)| \leq 1$.
3. *If $(a_r, i, j) \in \mathsf{owners}(\omega, a)$ and $a \neq a_r$ then $a \notin \mathrm{dom}(\mathcal{S})$.*

The definition of oracle refinement in Figure 6 preserves well-formedness.

**Lemma 1.** *If $\mathcal{S} \vdash \omega$ $\mathsf{wf}$ and $\mathcal{S}, \omega \Downarrow \gamma$ then $\mathcal{S} \vdash \mathsf{refine}(\mathcal{S}, \omega, \gamma)$ $\mathsf{wf}$.*

Producing well-formed oracles alone is insufficient to guarantee convergence. Oracle refinement would never reach a consistent state if the predicted spill areas were incorrectly sized.

The definition of oracle refinement in Figure 6 predicts spill areas that are correctly sized with respect to the current grid.

**Lemma 2.** *If $\mathcal{S} \vdash \omega$ $\mathsf{wf}$ and $\mathcal{S}, \omega \Downarrow \gamma$ then $\gamma \models \mathsf{refine}(\mathcal{S}, \omega, \gamma)$.*

Predicting correctly sized spill areas is also insufficient to guarantee convergence. Oracle refinement would never reach a consistent state if it oscillates between permitting and rejecting the same root to spill. Consider the sheet:

$$\text{Let } \mathcal{S} \stackrel{\mathsf{def}}{=} [\mathrm{A1} \mapsto \{1;2\}, \mathrm{B1} \mapsto \mathsf{IF}(\mathrm{A2} = 2, \{3;4\}, 0)]$$

Spill iteration would continue indefinitely if refinement cycled between the following two well-formed and correctly sized oracles:

$$[\mathrm{A1} \mapsto (2, 1, \checkmark)] \longrightarrow [\mathrm{A1} \mapsto (2, 1, \times), \mathrm{B1} \mapsto (2, 1, \checkmark)] \longrightarrow \cdots$$

To avoid oscillating spill iteration the process of oracle refinement should be *permit preserving*, defined below:

**Definition 2 (Permit preserving extension).** *We write $\gamma \vdash \omega \lesssim \omega'$ if oracle $\omega'$ is a permit preserving extension of $\omega$ in context $\gamma$. Defined as:*

$$\gamma \vdash \omega \lesssim \omega' \stackrel{\mathsf{def}}{=} \forall a, m, n, p.\ (\gamma \models_a \omega \wedge \omega(a) = (m, n, p)) \Rightarrow \omega'(a) = (m, n, p)$$

The definition of oracle refinement in Figure 6 is permit preserving.

**Lemma 3.** *If* $\mathcal{S} \vdash \omega$ wf *and* $\mathcal{S}, \omega \Downarrow \gamma$ *then* $\gamma \vdash \omega \lesssim \mathsf{refine}(\mathcal{S}, \omega, \gamma)$*.*

Spill iteration should be a converging iteration but this cannot be guaranteed in general; at any given step in the iteration a sheet can fail to evaluate to a grid. This can happen because the sheet contains a cell cycle, spill cycle, or diverging grid calculus term. Instead, we only expect that if the sheet is free from these divergent scenarios then spill iteration must converge. To allow us to dissect different forms of divergence and focus on spill iteration we only consider *acyclic* sheets, defined below:

**Definition 3 (Acyclic).** *A sheet* $\mathcal{S}$ *is acyclic if for all* $\omega$ *such that* $\mathcal{S} \vdash \omega$ wf*, there exists some* $\gamma$ *such that* $\mathcal{S}, \omega \Downarrow \gamma$*.*

For instance, none of the following sheets are acyclic: [A1 $\mapsto$ A1] has a cell cycle, [A1 $\mapsto$ B1 : C1] has a spill cycle, and [A1 $\mapsto$ $\Omega$] has a formula $\Omega$ that diverges. Divergent terms are not encodable in the spill calculus but are encodable in the grid calculus, as we show in Section 6.1. An alternative approach would be to explicitly model divergence in our semantics of sheet evaluation and show that iteration converges or the sheet diverges. We choose not to pursue this approach to improve the clarity of our operational semantics, but note that our semantics can be extended to model cycles.

For any acylic sheet, spill iteration will converge to a final spill oracle.

**Theorem 1 (Convergence).** *For all acyclic* $\mathcal{S}$ *and* $\omega$ *such that* $\mathcal{S} \vdash \omega$ wf*, there exists an oracle* $\omega'$ *such that* $\omega \longrightarrow^{*}_{\mathcal{S}} \omega'$ *and* $\mathcal{S} \vdash \omega'$ final.

*Proof.* (Sketch—see Appendix B of the extended version [21] for the full proof.) The value of any address with a binding is a function of its dependencies and the oracle prediction for that address. We inductively define an address as *fixed* if the oracle prediction is consistent for the address, and every address in the spill-dependency set (defined in [21]) is fixed. Lemma 3 states that correct predictions are always preserved, therefore a fixed address remains fixed through iteration and its value remains invariant. The dependency graph of the sheet is acyclic therefore if there is a non-fixed address then there must be a non-fixed address with no dependencies but an inconsistent oracle prediction—we call this a *non-fixed source*. Lemma 2 states that every new oracle correctly predicts the size with respect to the previous grid, therefore any non-fixed sources will be fixed in the new oracle. We conclude by observing that the number of fixed addresses in the sheet strictly increases at each step, and when every address is fixed the oracle is final.

### 4.6 Limitations and Differences with Real Systems

Permit preservation requires that if the size of an array does not change then the permit (which may be $\times$) is preserved—this property is crucial for our proof of convergence.

Real spreadsheet systems such as Sheets and Excel do not guarantee permit preservation. A root $a$ that is prevented from spilling using a permit $\times$ can later be permitted to spill, even if the size of the associated array does not change. This particular interaction arises when a root that was previously preventing $a$ from spilling changes dimension, freeing a previously occupied spill area. Permitting roots to spill into newly freed regions of the grid is desirable from a user perspective because it reflects the visual aspect of spreadsheet programming where an array will spill into any unoccupied cells.

A limitation of our formalism, if implemented directly, is that there exist some spreadsheets that when evaluated will prevent an array from spilling, despite the potential spill area being blank. Consider the sheet:

$$[\text{A3} \mapsto \{1, 2, 3\}, \text{C1} \mapsto \mathsf{IF}(\mathsf{ISERROR}(\text{A3}), 0, \{4; 5; 6\})]$$

When the total ordering used by oracle refinement orders A3 before C1 then the behaviour is as expected: A3 spills to the right and C1 evaluates to an error value. When the total ordering used by oracle refinement orders C1 before A3 then the behaviour appears peculiar: A3 evaluates to an error value and C1 evaluates to 0. The root A3 is prevented from spilling despite there appearing room in the grid! The issue is that the array in A3 never changes size, therefore the permit $\times$ assigned to the root is preserved, despite root C1 relinquishing the spill area on subsequent spill iterations.

The fundamental problem is one of constraint satisfaction. We would like to find a well-formed oracle that maximizes the number of roots that can spill in a deterministic manner. The total order on addresses ensures determinism but restricts the solution space. Our approach could be modified to deterministically permute the ordering until an optimal solution is found, however such a method would be prohibitively expensive.

Both Sheets and Excel find the best solution to our example sheet. We expect their implementations do not permute a total order on addresses, but implement a more efficient algorithm that runs for a bounded time. Finding a more efficient algorithm that is guaranteed to terminate remains an open challenge.

The limitation we present in our formalism only arises when a spreadsheet includes dynamic spill collisions and conditional spilling. We anticipate that this is a rare use case for spilled arrays, and does not arise when using spilled arrays to implement gridlets for live copy-paste-modify.

## 5 Grid Calculus: Spill Calculus with Sheets as Values

In this section we present the grid calculus: a higher-order spreadsheet calculus with sheets as values. The grid calculus extends the spill calculus of Section 4.

### 5.1 Extending Spreadsheets with Gridlets

The gridlet concept [12] has been proposed but not implemented. Our observation is that spilling a range reference acts much like copy-paste, but lacks local modification. We propose to implement gridlets using spilled arrays, by extending the spill calculus with primitives that implement first-class grid modification.

| | A | B | C |
|---|---|---|---|
| 1 | "*Edge*" | "*Len.*" | |
| 2 | "*a*" | 3 | B2^2 |
| 3 | "*b*" | 4 | B3^2 |
| 4 | "*c*" | SQRT(C4) | C2 + C3 |
| | ⋮ | ⋮ | ⋮ |

Source range A1:C4

| | A | B | C |
|---|---|---|---|
| | ⋮ | ⋮ | ⋮ |
| 6 | G(A1:C4, B2, 7, B3, 24) | | |
| 7 | | | |
| 8 | | | |
| 9 | | | |

Gridlet invocation in A6

Revisiting the example from the introduction, there are four key interactions happening in the invocation of a gridlet.

First, select the content in the grid that is to be modified.
Second, apply the selected modifications or updates.
Third, calculate the grid using the modified content.
Fourth and finally, project the calculated content into the grid.

Spreadsheets with spilled arrays support the final step but lack the capabilities to support the first three. We add these capabilities using four new constructs.

First-class sheet values $\langle \mathcal{S} \rangle$.
Operator GRID that evaluates to the current sheet.
Operator UPDATE that binds a formula in a sheet-value.
Operator VIEW that evaluates a given range in a sheet-value to an array.

Using these constructs we can implement gridlets, for example:

$$\mathsf{G}(\mathrm{A1{:}C4}, \mathrm{B2}, 7, \mathrm{B3}, 24) \stackrel{\mathsf{def}}{=} \\ \mathsf{VIEW}(\mathsf{UPDATE}(\mathsf{UPDATE}(\mathsf{GRID}, \mathrm{B2}, 7), \mathrm{B3}, 24), \mathrm{A1{:}C4})$$

Formatting is a core feature of Gridlets, but we omit formatting from the grid calculus for clarity, on the basis that it would be a straightforward addition. We now describe the details of the grid calculus.

## 5.2 Syntax and Operational Semantics

Figure 7 presents the syntax and operational semantics for the grid calculus. The grid calculus does not require modification of existing rules; we only add formula evaluation rules for the new constructs, and evaluation relations for *views*.

*Syntax* Let $x$ range over formula identifiers. Let $F$ range over formulas which may additionally be identifiers $x$, $\mathsf{LET}(x, F_1, F_2)$ which binds the result of evaluating $F_1$ to $x$ in $F_2$, GRID which captures the current sheet, $\mathsf{UPDATE}(F_1, a, F_2)$ which updates a formula binding in a sheet-value, and $\mathsf{VIEW}(F, r)$ which extracts a dereferenced range from a sheet-value. Let $V$ range over values which may additionally be a sheet-value $\langle \mathcal{S} \rangle$. Let $\mathcal{V}$ range over views; a view is a sheet with a range, denoted $(\mathcal{S}, r)$. A view range $r$ delimits the addresses to be computed in sheet $\mathcal{S}$.

Identifier $x \in$ IDENT
Formula $F ::= \cdots \mid x \mid \mathsf{LET}(x, F_1, F_2) \mid \mathsf{GRID} \mid \mathsf{UPDATE}(F_1, a, F_2) \mid \mathsf{VIEW}(F, r)$
Value $V ::= \cdots \mid \langle \mathcal{S} \rangle$
View $\mathcal{V} ::= (\mathcal{S}, r)$

Formula evaluation: $\mathcal{S}, \omega \vdash F \Downarrow V, \mathcal{D}$

$$\frac{\mathcal{S}, \omega \vdash F_1 \Downarrow V_1, \mathcal{D}_1 \qquad \mathcal{S}, \omega \vdash F_2[x := V_1] \Downarrow V_2, \mathcal{D}_2}{\mathcal{S}, \omega \vdash \mathsf{LET}(x, F_1, F_2) \Downarrow V_2, \mathcal{D}_1 \cup \mathcal{D}_2} \qquad \frac{}{\mathcal{S}, \omega \vdash \mathsf{GRID} \Downarrow \langle \mathcal{S} \rangle, \varnothing}$$

$$\frac{\mathcal{S}, \omega \vdash F_1 \Downarrow \langle \mathcal{S}_1 \rangle, \mathcal{D}}{\mathcal{S}, \omega \vdash \mathsf{UPDATE}(F_1, a, F_2) \Downarrow \langle \mathcal{S}_1[a \mapsto F_2] \rangle, \mathcal{D}_1} \qquad \frac{\mathcal{S}, \omega \vdash F \Downarrow \langle \mathcal{S}_1 \rangle, \mathcal{D} \qquad (\mathcal{S}_1, r) \Downarrow V}{\mathcal{S}, \omega \vdash \mathsf{VIEW}(F, r) \Downarrow V, \mathcal{D}}$$

View evaluation: $\mathcal{V}, \omega \Downarrow \gamma$

$$(\mathcal{S}, r), \omega \Downarrow \gamma \stackrel{\text{def}}{=} \forall a \in \mathrm{dom}(\mathcal{S}) \cap \mathsf{area}(r).\ \mathcal{S}, \omega \vdash a \,!\, \gamma(a)$$

Spill iteration: $\omega \longrightarrow_{\mathcal{V}} \omega'$

Final oracle: $\mathcal{V} \vdash \omega$ final

$$\frac{(\mathcal{S}, r), \omega \Downarrow \gamma \qquad \gamma \not\models \omega \qquad \mathsf{refine}(\mathcal{S}, \omega, \gamma) = \omega'}{\omega \longrightarrow_{(\mathcal{S}, r)} \omega'} \qquad \frac{\mathcal{V}, \omega \Downarrow \gamma \qquad \gamma \models \omega}{\mathcal{V} \vdash \omega \ \mathsf{final}}$$

Final view evaluation: $\mathcal{V} \Downarrow V$

$$(\mathcal{S}, r) \Downarrow V \stackrel{\text{def}}{=} [] \longrightarrow^{*}_{(\mathcal{S}, r)} \omega \text{ and } (\mathcal{S}, r) \vdash \omega \ \mathsf{final} \text{ and } \mathcal{S}, \omega \vdash r \Downarrow V, \mathcal{D}$$

**Fig. 7.** Syntax and Operational Semantics for Grid Calculus (Extends Figures 3—6)

*Formula evaluation:* $\mathcal{S}, \omega \vdash F \Downarrow V, \mathcal{D}$ A formula $\mathsf{LET}(x, F_1, F_2)$ evaluates in the standard way. A formula $\mathsf{GRID}$ evaluates to a sheet-value that captures the current sheet. A formula $\mathsf{UPDATE}(F_1, a, F_2)$ updates a formula binding in a sheet-value. If evaluating formula $F_1$ produces sheet-value $\langle \mathcal{S}_1 \rangle$ then $\mathsf{UPDATE}(F_1, a, F_2)$ evaluates to the sheet-value where $a$ is bound to $F_2$ in $\mathcal{S}_1$, denoted $\langle \mathcal{S}_1[a \mapsto F_2] \rangle$. A formula $\mathsf{VIEW}(F, r)$ evaluates a sheet-value and extracts a range. If evaluating formula $F$ produces sheet-value $\langle \mathcal{S}_1 \rangle$ then $\mathsf{VIEW}(F, r)$ evaluates to the value obtained by evaluating view $(\mathcal{S}_1, r)$. View evaluation is defined in Figure 7 and we describe the semantics at the end of the section. Here we address a subtle property of $\mathsf{VIEW}$; evaluating a view $(\mathcal{S}, r)$ adds no dependencies to the containing formula. Dependency tracking in our semantics is used to prevent spill cycles and captures dependence between *values* of addresses: the value of a spill root should not depend on the value of an address in the spill area. In contrast, sheet-values depend on the *formula* of an address in the containing sheet, but not the value of an address in the containing sheet. For example:

$$\text{Let } \mathcal{S} \stackrel{\mathsf{def}}{=} [\mathrm{A1} \mapsto \mathsf{VIEW}(\mathsf{UPDATE}(\mathsf{GRID}, \mathrm{A1}, 10), \mathrm{A2}), \mathrm{A2} \mapsto \mathrm{A1}]$$

Sheet $\mathcal{S}$ evaluates to grid $[\mathrm{A1} \mapsto 10, \mathrm{A2} \mapsto 10]$. What are the dependencies of each address? The value of A2 in the grid depends on the value of A1 in the grid. In contrast, the value of A1 in the grid does not depend on the value of A2 in the grid. This is because evaluating the formula in A1 constructs a private grid from which the value of A2 is obtained. However, A1 does depend on the formula of A2 in the containing grid. Our semantics only considers value dependence, therefore the dependency set of A1 is $\varnothing$—the address has no dependence on values in the containing grid.

Formula dependence is vital for efficient recalculation, though we do not model that in our semantics and only use dependency tracking to prevent spill cycles. If an address depends on the value of another address bound in a sheet, then it also depends on the formula of that address. The converse is not true in the presence of sheet-values.

*View evaluation:* $\mathcal{V}, \omega \Downarrow \gamma$ Evaluation of view $(\mathcal{S}, r)$ with oracle $\omega$ is defined in a similar manner as evaluation of sheets, however the induced grid $\gamma$ is limited to the sheet bindings that intersect the range $r$. There are two key consequences that arise from limiting the induced grid. First, we only evaluate the bindings in $\mathcal{S}$ required to evaluate the bindings in $r$. Second, only roots that are within range $r$ are permitted to spill; any root that is outside $r$ remains as an address containing a collapsed array. There is a difference between an address that holds a collapsed array and a root that is prevented from spilling an array by permit $\times$. The former has a pre-spill and post-spill value that is an array; the latter has a pre-spill value that is an array and a post-spill value that is an error.

*Spill iteration:* $\omega \longrightarrow_{\mathcal{V}} \omega'$ The definition of spill iteration for views is the same as spill iteration for sheets, except that we use view evaluation rather than sheet evaluation.

*Final oracle:* $\mathcal{V} \vdash \omega\ \mathsf{final}$ The definition of a final oracle for views is the same as a final oracle for sheets, except that we use view evaluation rather than sheet evaluation.

*Final view evaluation:* $\mathcal{V} \Downarrow V$ Evaluating a view $(\mathcal{S}, r)$ computes a final oracle for the view and then evaluates range $r$ in the context of sheet $\mathcal{S}$. Final view evaluation will evaluate range $r$, rather than extracting values from an induced grid, because viewing a range should sample all values in the range—including blank cells. If we extract values from the induced grid we can only obtain the values for addresses with a binding in $r$.

### 5.3 Formulas for Gridlets

We can encode the $\mathsf{G}$ operator using primitives from the grid calculus.

$$\llbracket \mathsf{G}(r, a_1, V_1, \ldots, a_n, V_n) \rrbracket = \mathsf{VIEW}(\llbracket (a_1, V_1, \ldots, a_n, V_n) \rrbracket, r)$$
$$\llbracket (a_1, V_1) \rrbracket = \mathsf{UPDATE}(\mathsf{GRID}, a_1, V_1)$$
$$\llbracket (a_1, V_1, \ldots, a_{n+1}, V_{n+1}) \rrbracket = \mathsf{UPDATE}(\llbracket (a_1, V_1, \ldots, a_n, V_n) \rrbracket, a_{n+1}, V_{n+1})$$

The $\mathsf{G}$ operator translates to the $\mathsf{VIEW}$ operator, and any bindings translate to a sequence of $\mathsf{UPDATE}$ operations. The initial sheet-value is obtained from the context using the $\mathsf{GRID}$ operator.

The translation illustrates that $\mathsf{G}$ is not higher-order because every application returns the value obtained by evaluating a view on a sheet-value. A language that only provides $\mathsf{G}$ does not permit sheet-values to escape and be manipulated by formulas. This is acceptable when emulating copy-paste because a copy is always taken with respect to the top-level sheet, however this does limit the usefulness of $\mathsf{G}$ as an implementation construct. This limitation motivates the design of the grid calculus; as we show in the next section, the grid calculus is capable of encoding other language features.

## 6 Encoding Objects, Lambdas, and Functions

In this section we give three encodings that target the grid calculus: objects, lambdas, and sheet-defined functions.

### 6.1 Encoding the Abadi and Cardelli Object Calculus

We introduce the grid calculus to implement gridlets and the concept of live copy-paste. Perhaps surprisingly, the grid calculus can encode object-oriented programming, in particular the untyped object calculus of Abadi and Cardelli [1]. Their calculus is a tiny object-based programming language, akin to a prototype-based language such as Self [6], but capable of representing class-based object-oriented programming via encodings.

We draw a precise analogy between spreadsheets and objects. A sheet is like an object. A cell is like a method name. A formula in a cell is like a method implementation. The $\mathsf{GRID}$ operator is like the this keyword. Formula update is like method update.

We assume an isomorphism between method names $\ell$ and cell addresses $a$ and use $\ell$ in both the object calculus and grid calculus. We define the translation of object calculus terms to grid calculus formulas, denoted $\llbracket b \rrbracket$, as follows:

$$\llbracket x \rrbracket = x$$
$$\llbracket [\ell_i = \varsigma(x_i) b_i{}^{i \in 0..n}] \rrbracket = \langle [\ell_i \mapsto \llbracket \varsigma(x_i) b_i \rrbracket^{i \in 0..n}] \rangle$$
$$\llbracket b.\ell \rrbracket = \mathsf{VIEW}(\llbracket b \rrbracket, \ell)$$
$$\llbracket b_1.\ell \Leftarrow \varsigma(x) b_2 \rrbracket = \mathsf{UPDATE}(\llbracket b_1 \rrbracket, \ell, \llbracket \varsigma(x) b_2 \rrbracket)$$
$$\llbracket \varsigma(x) b \rrbracket = \mathsf{LET}(x, \mathsf{GRID}, \llbracket b \rrbracket)$$

The translation makes our analogy concrete. We use the LET formula to lexically capture *self* identifiers. The grid calculus allows the construction of diverging formulas, as discussed in Section 4.5. We demonstrate this using a diverging object calculus term.

$$\Omega = [\![[\mathrm{A1} = \varsigma(x)x.\mathrm{A1}].\mathrm{A1}]\!] = \mathsf{VIEW}(\langle[\mathrm{A1} \mapsto \mathsf{LET}(x, \mathsf{GRID}, \mathsf{VIEW}(x, \mathrm{A1}))]\rangle, \mathrm{A1})$$

The operational semantics are preserved by the translation. We assume a big-step relation for object calculus terms, denoted $b \Downarrow o$. The proof is in Appendix C of the extended version [21].

**Theorem 2.** *If $b$ is a closed and $b \Downarrow o$ then $[], [] \vdash [\![b]\!] \Downarrow [\![o]\!], \varnothing$.*

### 6.2 Encoding the Lambda Calculus

We give an encoding of the lambda calculus that is inspired by the object calculus embedding of the lambda calculus. We use ARG1 to hold the argument and VAL1 to hold the result of a lambda. In spreadsheet languages both ARG1 and VAL1 are legal cell addresses; for example, address ARG1 denotes the cell at column 1151 and row 1.

$$\begin{aligned} [\![x]\!] &= x \\ [\![\lambda x.M]\!] &= \mathsf{UPDATE}(\mathsf{GRID}, \mathrm{VAL1}, \mathsf{LET}(x, \mathsf{VIEW}(\mathsf{GRID}, \mathrm{ARG1}), [\![M]\!])) \\ [\![M\ N]\!] &= \mathsf{VIEW}(\mathsf{UPDATE}([\![M]\!], \mathrm{ARG1}, [\![N]\!]), \mathrm{VAL1}) \end{aligned}$$

### 6.3 Encoding Sheet-Defined Functions

A sheet-defined function [14, 17, 19, 20] is a mechanism for a user to author a function using a region of a spreadsheet. We can model a sheet-defined function $f$ as a triple $(\mathcal{S}, (a_0, \ldots, a_n), r)$ that consists of the moat or sheet-bindings for the function, the addresses from the moat that denote arguments, and the range from the moat that denotes the result. The application $f(V_0, \ldots, V_n)$ can be encoded in the grid calculus as follows, where $f = (\mathcal{S}, (a_0, \ldots, a_n), r)$:

$$\begin{aligned} [\![f(V_0, \ldots, V_n)]\!] &= \mathsf{VIEW}([\![(V_0, \ldots, V_n)]\!], r) \\ [\![()]\!] &= \langle \mathcal{S} \rangle \\ [\![(V_0, \ldots, V_{n'+1})]\!] &= \mathsf{UPDATE}([\![(V_0, \ldots, V_{n'})]\!], a_{n'+1}, V_{n'+1}) \end{aligned}$$

## 7 Related Work

*Formal Semantics of Spreadsheets.* Our core calculus is similar to previous formalisms for spreadsheets, Several previous works [3, 7, 14, 19] offer formal semantics for spreadsheet fragments. Mokhov et al. [16] capture the logic of recalculating dependent cells. Finally, Bock et al. [4] provide a cost semantics for evaluation of spreadsheet formulas.

*Spilling.* Major spreadsheet implementations like Sheets [6] and Excel [7] implement spilled arrays [11], but do not document details of the implementation. In [17], authors propose a spilling-like mechanism that allows matrix values in cells to spread across a predefined range—this is closely related to *"Ctrl+Shift+Enter" formulas* [8] in Excel. The proposal in [17] is significantly simpler than spilled arrays because the dimension of the spilled area is fixed and declared ahead of time. Sarkar et al. [18] note that spilled arrays violate Kay's *value principle* [13] because a user is unable to edit constituent cells, except for the spill root.

*Extending the Spreadsheet Paradigm.* Clack and Braine [8] propose a spreadsheet based on a combination of functional and object-oriented programming. Their integration is different from our analogy: in their system, a class is a collection of parameterised worksheets, and a parameterised worksheet corresponds to a method. In gridlets, the grid corresponds to an object and cells on the grid correspond to methods of the object.

*Similarity Inheritance in Forms/3.* Forms/3 [5] is a visual programming language that borrows the key concept of cell from spreadsheets. Instead of a tabular sheet, cells in Forms/3 are arranged on a *form*: a canvas with no structure. Forms/3 explored an abstraction model called "similarity inheritance" through which a form may borrow cells from another form and optionally modify attributes of certain cells. This resembles substitution in gridlets, however reusing a portion of the tabular grid and spilling into adjacent cells are primary to gridlets, whereas such notions are absent from Forms/3.

*Sheet-defined Functions.* Sheet-defined functions [17] (SDFs) allow the user to reuse logic defined using formulas in the grid. The user nominates input cells, an output cell, and gives the function a name. When the function is called, a virtual copy of the workbook is instantiated. Arguments to the function are placed in the input cells, the virtual workbook is calculated, and the result from the output cell is returned.

Elastic SDFs [14] generalize SDFs to handle input arrays of arbitrary size. In [4], the authors provide a precise semantics for SDFs, closures and array formulas, but not for spilling. Gridlets are more general than SDFs as each Gridlet invocation can have a unique set of local substitutions, whereas all calls to an SDF share the same arguments, giving greater flexibility to the user.

*Error prevention and Error detection.* Abraham and Erwig propose type systems for error detection [3] and automatic model inference [2]. Abraham and Erwig [3] provide an operational semantics for sheets that is similar to the core calculus in Section 3, but they do not give a semantics for spilled arrays.

Gencel [10] is a typed "template language" that describes the layout of a desired worksheet along with a set of customized update operations that are specific

[6] https://support.google.com/docs/answer/6208276?hl=en
[7] https://aka.ms/excel-dynamic-arrays
[8] https://aka.ms/excel-cse-formulas to the particular template. The type system guarantees that the restricted set of update operations keeps the desired worksheet free from omission, reference, and type errors.

Cheng and Rival [7] use abstract interpretation to detect formula errors due to mismatch in type. Their technique also incorporates analysis of associated programs, such as VBA scripts, along with formulas on the grid.

## 8 Conclusion

Repetition is common in programming—spreadsheets are no different. The distinguishing property of spreadsheets is that reuse includes formatting and layout, and is not limited to formula logic. Gridlets [12] are a high-level re-use abstraction for spreadsheets. In this work we give the first semantics of gridlets as a formula. Our approach comes in two stages.

First, we make sense of spilled arrays, a feature that is available in major spreadsheet implementations but not previously formalised. The concept is simple and belies the many subtleties involved in implementing spilled arrays. We present the spill calculus as a concise description of spilling in spreadsheets.

Second, we extend the spill calculus with the tools to implement gridlets. The grid calculus introduces the concept of first-class sheet values, and describes the semantics of three higher-order operators that emulate *copy-paste-modify*. The composition of these operators gives the semantics for gridlet operator $\mathsf{G}$.

Spreadsheet programming bears a resemblance to object-oriented programming, alluded to often in the literature. We show that the resemblance runs deep by giving an encoding of the object calculus into the grid calculus, with a direct parallel between objects and sheets.

**Acknowledgements**

Thank you to the Microsoft Excel team for hosting the second author during his research internship at Microsoft's Redmond campus. Thank you to Tony Hoare, Simon Peyton Jones, Ben Zorn, and members of the Microsoft Excel team for their feedback and assistance with this work.

Cambridge, UK, and Davis, California, USA
Spreadsheet Day, October 17, 2019

## References

[1] Abadi, M., Cardelli, L.: A Theory of Objects. Monographs in Computer Science, Springer (1996)

[2] Abraham, R., Erwig, M.: Inferring templates from spreadsheets. In: Proceedings of the 28th International Conference on Software Engineering. pp. 182–191. ICSE ’06, ACM, New York, NY, USA (2006)

[3] Abraham, R., Erwig, M.: Type inference for spreadsheets. In: Proceedings of the 8th ACM SIGPLAN International Conference on Principles and Practice of Declarative Programming. pp. 73–84. PPDP ’06, ACM, New York, NY, USA (2006)

[4] Bock, A.A., Bøgholm, T., Sestoft, P., Thomsen, B., Thomsen, L.L.: Concrete and abstract cost semantics for spreadsheets. Tech. Rep. TR-2008-203, IT University of Copenhagen (2018)

[5] Burnett, M., Atwood, J., Djang, R.W., Reichwein, J., Gottfried, H., Yang, S.: Forms/3: A first-order visual language to explore the boundaries of the spreadsheet paradigm. Journal of functional programming **11**(2), 155–206 (2001)

[6] Chambers, C., Ungar, D.M.: Customization: Optimizing compiler technology for self, A dynamically-typed object-oriented programming language. In: PLDI. pp. 146–160. ACM (1989)

[7] Cheng, T., Rival, X.: Static analysis of spreadsheet applications for type-unsafe operations detection. In: Vitek, J. (ed.) Programming Languages and Systems, pp. 26–52. Springer, Berlin Heidelberg, Berlin, Heidelberg (2015)

[8] Clack, C., Braine, L.: Object-oriented functional spreadsheets. In: 10th Glasgow Workshop on Functional Programming. pp. 1–12 (1997)

[9] Djang, R.W., Burnett, M.M.: Similarity inheritance: a new model of inheritance for spreadsheet vpls. In: Proceedings. 1998 IEEE Symposium on Visual Languages (Cat. No. 98TB100254). pp. 134–141. IEEE (1998)

[10] Erwig, M., Abraham, R., Cooperstein, I., Kollmansberger, S.: Automatic generation and maintenance of correct spreadsheets. In: Proceedings of the 27th international conference on Software engineering. pp. 136–145. ACM (2005)

[11] Jelen, B.: Excel Dynamic Arrays Straight to the Point. Holy Macro! Books (2018), see also https://blog-insider.office.com/2019/06/13/dynamic-arrays-and-new-functions-in-excel/

[12] Joharizadeh, N., Sarkar, A., Gordon, A.D., Williams, J.: Gridlets: Reusing spreadsheet grids. In: Extended Abstracts of the 2020 CHI Conference on Human Factors in Computing Systems. CHI EA ’20, ACM, New York, NY, USA (2020). 10.1145/3334480.3382806,http://doi.acm.org/10.1145/3334480.3382806

[13] Kay, A.: Computer software. Scientific American 251(3), 52–59 (1984), http://www.jstor.org/stable/24920344

[14] McCutchen, M., Borghouts, J., Gordon, A.D., Peyton Jones, S., Sarkar, A.: Elastic sheet-defined functions: Generalising spreadsheet functions to variable-size input arrays (2019), unpublished manuscript available at https://aka.ms/calcintel

[15] McCutchen, M., Itzhaky, S., Jackson, D.: Object spreadsheets: A new computational model for end-user development of data-centric web applications. In: Proceedings of the 2016 ACM International Symposium on New Ideas, New Paradigms, and Reflections on Programming and Software. pp. 112–127. Onward! 2016, ACM, New York, NY, USA (2016)

[16] Mokhov, A., Mitchell, N., Peyton Jones, S.: Build systems à la carte. PACMPL 2(ICFP), 79:1–79:29 (2018)

[17] Peyton Jones, S.L., Blackwell, A.F., Burnett, M.M.: A user-centred approach to functions in Excel. In: ICFP. pp. 165–176. ACM (2003)

[18] Sarkar, A., Gordon, A.D., Jones, S.P., Toronto, N.: Calculation view: multiple-representation editing in spreadsheets. In: 2018 IEEE Symposium on Visual Languages and Human-Centric Computing (VL/HCC). pp. 85–93 (Oct 2018). 10.1109/VLHCC.2018.8506584

[19] Sestoft, P.: Implementing function spreadsheets. In: Proceedings of the 4th international workshop on End-user software engineering. pp. 91–94. ACM (2008)

[20] Sestoft, P., Sørensen, J.Z.: Sheet-defined functions: Implementation and initial evaluation. In: Dittrich, Y., Burnett, M., Mørch, A., Redmiles, D. (eds.) End-User Development, pp. 88–103. Springer, Berlin Heidelberg, Berlin, Heidelberg (2013)

[21] Williams, J., Joharizadeh, N., Gordon, A.D., Sarkar, A.: Higher-order spreadsheets with spilled arrays (with appendices). Tech. rep., Microsoft Research (2020), https://aka.ms/calcintel

